# Cystic endosalpingiosis presenting as chronic back pain, a case report

**DOI:** 10.1186/1746-1596-8-196

**Published:** 2013-12-03

**Authors:** Andreas H Scheel, Josef Frasunek, Werner Meyer, Philipp Ströbel

**Affiliations:** 1 Department of Pathology, University Medical Center Göttingen, Göttingen, Germany; 2 Institute of Pathology Nordhessen, Kassel, Germany; 3 Department of Gynaecology, Helios Albert-Schweitzer-Klinik Northeim, Northeim, Germany

**Keywords:** Cystic endosalpingiosis, Endometriosis, Chronic back pain, Pelvic tumour

## Abstract

A 48-year old woman presented with chronic back pain. Previous examinations had been inconclusive. Gynaecological examination revealed large cystic masses on the fundus uteri and left adnexa. Laparoscopy and histopathology showed unusually extensive cystic endosalpingiosis covering the serosa-coated uterine surface as well as the adnexa on both sides. After uneventful laparoscopic-assisted vaginal hysterectomy the patient quickly recovered and was relieved of her chronic backache.

**Virtual slides:**

http://www.diagnosticpathology.diagnomx.eu/vs/1501709091077524.

## Background

Endosalpingiosis and the related condition endometriosis cause variable symptoms which frequently include chronic back pain [1]. Given that up to 10% of women suffer from endometriosis [2] the presented case illustrates the need to include gynaecological anamnesis in the clinical work-up of chronic back pain. Furthermore, cystic endosalpingiosis may initially be detected as suspicious cystic formation that has to be carefully differentiated from neoplasia.

## Case presentation

A 48-year old woman had cystic formations at the fundus uteri and left adnexa. The formations had been detected 2.5 years earlier by transvaginal ultrasound examination during routine gynaecological check-up. Given the innocent presentation of the findings and the absence of additional clinical symptoms the patient was kept under watchful surveillance. During the latest check-up, a slight increase in size of the cystic formation was noticed. The patient has two healthy children (1 vaginal partus, 1 caesarean section) and consented to hysterectomy. During the detailed anamneses the patient reported to have suffered from chronic back pain for a period of several years. Previous orthopaedic and neurologic examinations had been inconclusive.

### Clinical investigations and histopathology

**Figure 1:**
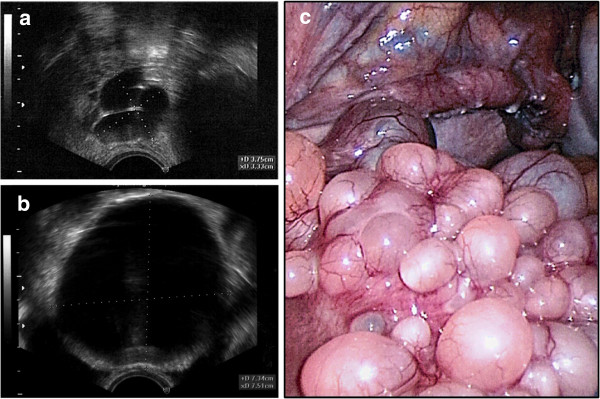
**Clinical presentation.** Transvaginal ultrasound showed cystic formations in the areas of the right fallopian tube **(a)** and fundus uteri **(b)**. The maximum diameter of the cysts was 7.5 cm. Laparoscopic presentation of the cystic mass **(c)**; uterus (front) with right tube and right abdominal wall (background).

**Figure 2:**
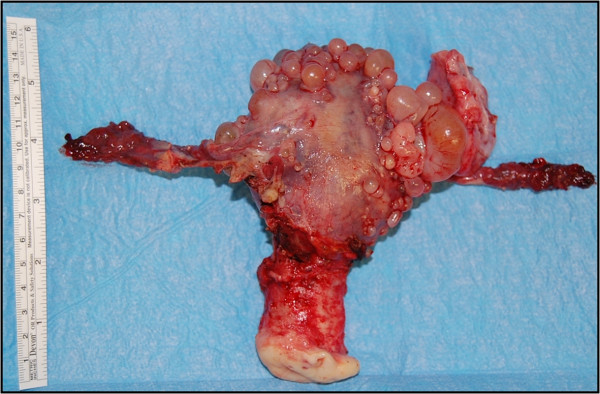
**Resected uterus including both fallopian tubes and the left ovary.** The serosa-coated surface is covered with endosalpingiosis cysts.

The transvaginal ultrasound examination showed cystic formations with multiple well delimited cysts at the fundus uteri and the adnexa (Figure 1a-b). The cysts had homogeneous, echo-free lumina and smooth walls. The maximum diameter was 7.5 cm. During laparoscopy, cystic formations were present on the surface of the fundus uteri and on both adnexa (Figure 1c). A biopsy was taken and histologically investigated by frozen sections. Neoplasia was ruled out and the uterus including both fallopian tubes and the left ovary were removed by laparoscopic-assisted vaginal hysterectomy (Figure 2).

**Figure 3:**
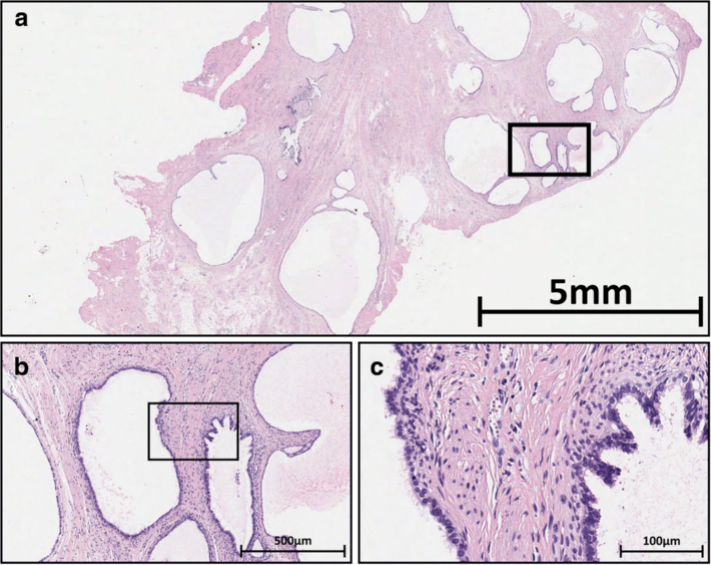
**The biopsy taking during laparoscopy shows cysts lined with cubic to cylindrical epithelium.** Endometrial stroma is absent (**a**, 11× magnification). High power magnifications (**b**: 100×, **c**: 400×) reveal that the well-differentiated epithelium has cilia, much like fallopian tubes epithelium (Black boxes: magnified areas).

Histology of the formalin-fixed specimen confirmed the diagnosis of cystic endosalpingiosis: the cysts were lined with single-layered prismatic epithelium without cellular atypia (Figure 3a-b). High-power magnification demonstrated cilia on the luminal aspect (Figure 3c). No endometrial stroma was present.

The patient quickly recovered from the hysterectomy and experienced a relief of her chronic back pain. Follow-up gynaecological check-ups detected no abnormalities.

### Differential diagnosis

Chronic back pain in women may be a symptom of endometriosis or endosalpingiosis especially during reproductive age. Clinical work-up of back pain should include gynaecological anamnesis. Vice versa, anamnesis in patients with known endometriosis should include back pain.

Cystic endosalpingiosis may present itself as large cystic mass mimicking neoplasia. Biopsy and histology may confirm the diagnosis and rule out malignancy should ultrasound and clinical examinations be inconclusive.

## Discussion

Endosalpingiosis and endometriosis are related benign conditions caused by ectopic epithelium of tubal-like or endometrial differentiation. They affect women mostly during reproductive age and a have a high prevalence of 5-10% [2]. As they cause variable symptoms overlapping with several other diseases, diagnosis can be challenging [1]. The presented case exemplifies chronic back pain in a 48 year old woman as symptom of extensive cystic endosalpingiosis.

Endosalpingiosis refers to the presence of ectopic epithelium with tubal differentiation featuring cilia in the small pelvis and lower abdominal cavity. Endometriosis on the other hand is characterised by ectopic, functional endometrium responding to estrogens and featuring endometrial stroma. Clinical and experimental evidence suggests two mechanisms of pathogenesis: Metastatic spread of epithelia to their ectopic place and metaplasia of the affected mesothelium. Both seem to occur: Studies demonstrating a relation between increased shedding of endometrial progenitor cells during menstruation and endometriosis argue for metastatic spread to be the predominant cause [3]. The anatomic distribution of endometriosis in the lower pelvis seems explainable by uterine position and gravity. Rare cases of metastatic spread to distant sites such as the thorax may be caused by different mechanisms [4,5]. On the other hand, the metaplastic potential of the pelvic mesothelium suggests metaplasia to be the most common origin of endosalpingiosis: the mesothelium of the pelvic and lower abdomen is ontogenetically related to the Müllerian ducts which give rise to the primary female genitals. Metaplasia may also explain endometriosis in certain cases such as obstructed fallopian tubes and in rare male endometriosis patients [6,7]. Metaplasia into uterine tissues is not limited to mesothelium as is exemplified by rare cases of endocervical glands in the urinary bladder [8]. Apparently urothelium may undergo metaplasia into endocervical glands without stroma; however, müllerian tissue displaced during embryonic development or during surgical interventions are possible alternative mechanism for such findings.

Endometriosis is symptomatic in most cases while endosalpingiosis is usually an incidental finding. Both condition may cause a varying spectrum of symptoms depending on the sites involved. The intensity of the symptoms is not directly related to the extent of the condition [9]. The most common symptoms include: acquired dysmenorrhoea, lower abdominal, pelvic and back pain, dyspareunia, irregular bleeding and infertility. Back pain was reported in up to 29% of cases [1] and infertility in up to 30% [10]. Obviously none of these symptoms are specific.

A detailed picture of the molecular alterations underlying endometriosis emerged highlighting increased local formation of prostagladin E2 and estradiol. Epigenetic changes may initially raise prostagladin and estrogen receptor β levels and lead to feedback loops sustaining the endometriosis and causing inflammation [4]. Accordingly hormone therapy and nonsteroidal anti-inflammatory drugs are deployed to treat endometriosis [1]. Medical therapy successfully controls symptoms in most cases yet has a high rate of recurrence. Surgical rehabilitation is another option, especially in cases of infertility linked to endometriosis [1]. In general, the treatment of endometriosis is complicated and protracted.

Endosalpingiosis may become symptomatic by mechanical irritation of abdominal organs. In cases of extensive cystic endosalpingiosis surgical removal may effectively abolish symptoms [11-13]. The presented case exemplifies the diagnostic challenge of endosalpingiosis and endometriosis: A 48-year old woman had been under watchful surveillance for a uterine cystic mass for 2.5 years. She also suffered from chronic back pain yet the two symptoms were not related- from the patient's point of view, there was no connection and she only mentioned the back pain to her gynaecologist upon detailed anamnesis. After uncomplicated laparoscopic-assisted vaginal hysterectomy she was relieved of her symptoms. A complete relief of the symptoms could not have been guaranteed yet the hysterectomy could have been performed earlier given the patient has two healthy children and intends no further family planning.

Endosalpingiosis forming cysts with diameters of several centimetres are rare findings described in the literature as single case reports [11-13] or small series of patients [14]. The usual macroscopic presentation features tiny cysts with few millimetres diameter covering the surface of the uterus or lining the small pelvis. The cysts may give the ectopic epithelium a suspicious papillary appearance.

Endosalpingiosis, as well as endometriosis may occasionally be encountered in unlikely anatomic regions such as the thorax or even axillary lymph nodes [5,15]. Experimental evidence indicates displacement of detached epithelium via lymphatic or blood vessels as possible mechanism. Intravenous injection of endometrium could induce pulmonary endometriosis in rabbits [7].

Histologically the metaplastic epithelia are clearly distinguishable from neoplasia by their lack of mitoses, lack of cellular atypia and absence of desmoplastic stroma. The crux is to keep these benign conditions in mind both in clinical practice and in surgical pathology.

## Conclusions

Endometriosis and endosalpingiosis are benign conditions in woman caused by ectopic endometrial or tubal-type epithelium. They may cause a broad spectrum of symptoms including back pain. Given their high prevalence of up to 10% of woman they are important differential diagnoses in non-distinctive pelvic pain and backache. While endometriosis is symptomatic in most cases, endosalpingiosis is usually an incidental finding.

Though the ectopic epithelium will not undergo malignant transformation it might form large masses mimicking neoplasia. Ectopic epithelia are occasionally found in distant anatomic sites such as lymph nodes. The histological finding are distinctly benign yet the macroscopic impression and general presentation may be puzzling.

Remedial actions may ameliorate or abolish symptoms yet in general the treatment of endometriosis is complicated and protracted. Endosalpingiosis on the other hand usually does not require treatment as it is usually asymptomatic and never undergoes transformation.

## Consent

Written informed consent was obtained from the patient for publication of this Case Report and any accompanying images. A copy of the written consent is available for review by the Editor-in-Chief of this journal.

## Competing interests

The authors declare no competing interests.

## Authors’ contributions

The patient was clinically seen and treated by JF. JF is director of the department of Gynaecology and Obstetrics at the Helios Clinic Northeim, Germany. Histopathology of the specimens was performed by WM and AHS under the guidance of PS in the Department of Pathology of the University Medical Center Göttingen, Germany. PS is director of the Department. The manuscript was drafted by AHS and critically reviewed and discussed with the co-authors. JF recorded ultrasound images and photographs of the macroscopic specimens. AHS and WM prepared the microphotographs. All authors read and approved the final manuscript
